# Nanoscale Zero-Valent Iron Has Minimum Toxicological Risk on the Germination and Early Growth of Two Grass Species with Potential for Phytostabilization

**DOI:** 10.3390/nano10081537

**Published:** 2020-08-05

**Authors:** Manuel Teodoro, Rafael Clemente, Ermengol Ferrer-Bustins, Domingo Martínez-Fernández, Maria Pilar Bernal, Martina Vítková, Petr Vítek, Michael Komárek

**Affiliations:** 1 Department of Environmental Geosciences, Faculty of Environmental Sciences, Czech University of Life Sciences Prague, Kamýcká 129, 16500 Praha–Suchdol, Czech Republic; teodoro@fzp.czu.cz (M.T.); domingo.marfer@gmail.com (D.M.-F.); vitkovam@fzp.czu.cz (M.V.); 2 Department of Soil and Water Conservation and Organic Waste Management, CEBAS-CSIC, Campus Universitario de Espinardo, 30100 Murcia, Spain; rclemente@cebas.csic.es (R.C.); pbernal@cebas.csic.es (M.P.B.); 3 Escola Politécnica Superior, Universitat de Girona, Carrer de Maria Aurèlia Capmany i Farnés 61, 17003 Girona, Spain; ermen.ferrer16@gmail.com; 4 Global Change Research Institute, Czech Academy of Sciences, Bělidla 986/4a, 60300 Brno, Czech Republic; vitek.p@czechglobe.cz

**Keywords:** nano zerovalent iron, plant stress, uptake, *Agrostis capillaris*, *Festuca rubra*

## Abstract

Two *Poaceae* species, *Agrostis capillaris* and *Festuca rubra*, were selected for their potential as phytostabilizing plants in multicontaminated soils. These species are resistant to contamination and maintain high concentrations of contaminants at the root level. Nanoscale zero-valent iron (nZVI) is an engineered nanomaterial with the ability to stabilize metal(loid)s in soils; its potential toxicological effects in the selected species were studied in a germination test using: (i) control variant without soil; (ii) soil contaminated with Pb and Zn; and (iii) contaminated soil amended with 1% nZVI, as well as in an hydroponic experiment with the addition of nZVI 0, 25, 50 and 100 mg L^−1^. nZVI had no negative effects on seed germination or seedling growth, but was associated with an increase in shoot growth and reduction of the elongation inhibition rate (root-dependent) of *F. rubra* seedlings. However, applications of nZVI in the hydroponic solution had no effects on *F. rubra* but *A. capillaris* developed longer roots and more biomass. Increasing nZVI concentrations in the growing solution increased Mg and Fe uptake and reduced the Fe translocation factor. Our results indicate that nZVI has few toxic effects on the studied species.

## 1. Introduction

Some of the adverse effects caused by soil contamination can be decreased by chemical stabilization, which implies the addition of materials to soil to decrease the mobility and bioavailability of metallic and metalloid contaminants [1,2]. In past years, engineered nanoparticles (ENP), materials with at least two dimensions between 1 and 100 nm, have played a role in newly developed remediation technologies [3,4,5]. These ENPs can function as scavengers for contaminants, mainly because of their high reactivity and large specific surface area [6]. Nanosized zero-valent iron (nZVI) has recently become popular for use as a stabilizing agent for metal and metalloid contaminants, due to its strong reducing properties and high reactivity, which affect contaminant mobility through adsorption, redox reactions and surface precipitation and/or co-precipitation in the form of metal iron oxides [7,8,9,10].

Stabilization of metal (loid) contaminants can be considered one of the most realistic and viable alternatives for the recovery and conservation of heavily contaminated soils [11]. The use of immobilizing amendments in general, including nZVI, can decrease contaminant solubility (e.g., risk of ground water contamination) and bioavailability [12]. This is an important factor for establishing vegetation cover that can lead to ecological restoration. Phytostabilization is an efficient remediation approach that includes establishing plant cover by using species that can stabilize pollutants in the root zone (e.g., roots and rhizosphere) by accumulation or precipitation and, thus, reduce their mobility and bioaccessibility [13,14]. Hence, species that are tolerant to high contamination levels and, at the same time, present an extensive fasciculated root system, rapid growth and establishment, high longevity, easy maintenance, good adaptation to contamination and below-ground accumulation of metals are the best candidates for phytostabilization [14,15,16]. The selection of the appropriate amendment or combination of amendments is also crucial for the success of the remediation process [1,14,17]. The interactions between the selected amendment and plant species and the subsequent relationship with the target contaminants will condition the restoration process. A positive response of these interactions in terms of soil restoration may help phytostabilization to being successfully assisted by the amendment. Therefore, the understanding of such interactions has recently gained substantial attention [1,18,19,20].

The use of ENPs may lead to their accumulation in edible parts of plants and the appearance of adverse effects on agronomic traits [21]. Most environmentally relevant ENPs have toxic effects in plants at relatively high concentrations, and the toxicity is usually species-dependent [21,22]. However, some plants have shown signs of recuperation, thus indicating that the toxicity was temporary [23]. Due to its high reactivity, nZVI accumulates easily in the environment and is vulnerable to oxidation [24]. Most studies related to nZVI interactions with plants have shown no or minimal (usually at high concentrations) effects on their functions, e.g., Ma, et al. [25] found a reduction of transpiration and growth of poplars after the application of 200 mg L^−1^ nZVI; Martínez-Fernández and Komárek [23] found reductions in the root hydraulic conductivity of tomato plants growing at concentrations of 100 mg L^−1^ nZVI, due to aggregation on the root surfaces. These works suggest that alterations of plant functions may not be due simply to direct chemical phytotoxicity, but are also a result of physical interactions between ENPs and plant cell transport paths [7,8,9,10,11].

The inhibition of seed germination and seedling growth are among the most evident effects of toxic compounds on plants [26], several studies have reported direct influence of ENP on seed germination, both positive and negative [22,24,27,28], however, few have shown the effects under the interaction of both, ENP and contaminants. Hydroponic systems provide a certain degree of control on nutrient concentrations and other molecules provided to the plant and they allow less invasive separations of root and shoot tissues for analysis [29]. Moreover, the influence of reactions in the soil system are omitted, which makes it possible to investigate the direct interactions between roots and nutrients, as well as the effects of the ENPs at the root surfaces [23].

To investigate the potential toxicity caused by nZVI, a germination test and hydroponics experiment were conducted, using two plant species. Based on the literature review, we hypothesize that negative effects of nZVI on *Poaceae* species can be reflected in (a) low germination potential [24,28]; (b) the limitation of plant growth [21,22,23]; (c) the reduction of nutrient uptake [23,26]; (d) the production of stress-related amino acids, such as proline [30,31] and/or photosynthesis-related pigments, such as carotenoids [32,33]. Previous studies [34] have demonstrated the ecological value and phytostabilization potential of *Agrostis capillaris* L. and *Festuca rubra* L. in soil contaminated mostly with Pb and Zn. Therefore, in order to contribute to the knowledge of assisted phytostabilization and the search for ideal associations of plant-amendment, the interactions between nZVI and the mentioned species were investigated.

## 2. Materials and Methods

### 2.1. Sources of Soil and Seeds

For the germination experiment, soil contaminated with Pb and Zn was used [35,36]. The soil was collected from the mining and smelting area of Příbram, Czech Republic. The soil was obtained from 12 random sampling sites at two separate times (e.g., March and May, 2017). After collection, the samples were air dried, homogenized and sieved at 2 mm. Characterization of the soil properties can be found in Table 1. The nZVI used was an air-stable product (NANOFER STAR) produced by Nano Iron Ltd. (Židlochovice, Czech Republic).

Seeds of *A. capillaris* and *F. rubra* were collected from the contaminated site during March and May 2017 using transect sampling. The seeds were extracted directly from the spikes and were preserved in glass containers at 3 °C until their use in the experiment. Prior to the experiments with nZVI, several germination tests were run at different conditions of the substrate and stratification (cold, hot, acid and mechanical) with very little success (total germination < 10%). Therefore, in order to introduce a control variant for comparing the collected seeds, two sources of seeds were tested: (i) “commercial” seeds were purchased from a producer (Planta Naturallis, Markvartice, Czech Republic) and (ii) “native” seeds, obtained directly from the contaminated site. Soil fertility has direct influence on the production of the seeds, the availability of nutrients and contaminants during the production of the seeds influence the size, water content and their vigor [37].

### 2.2. Germination Experiment

Prior the experiment, the seeds were disinfected using 30% H_2_O_2_, and then placed in petri dishes inside a germination chamber at 20 °C and 65% relative humidity under a 16:8 h period of light/darkness. The experiment was separated in two parts for practical reasons explained above: first the control, where 50 seeds were sown using filter paper as a substrate (10 replicates); secondly, the study with nZVI where 20 seeds were sown (6 replicates) using as the substrate the contaminated soil or using contaminated soil treated with 1% nZVI after one month of stabilization. Several studies [8,10,25,36] have shown that applications of 1% of nZVI into contaminated soil can successfully reduce the mobilization of metals and metalloids.

The total number of germinated seeds was recorded daily starting from the 6th day. After 20 days, the seedlings were harvested and root and shoot lengths were measured. The calculations performed include the relative germination rate (*RGR*, Equation (1)), which is defined as the ratio of the total number of germinated seeds in the treated soils (*Gs*) and in the control (*Gc*):

*RGR = Gs/Gc* (1)

The elongation inhibition rate (*EIR*, Equation (2)) is defined as the difference between the root lengths in the control (*Lc*) and in the treated soils (*Ls*), using the formula:

*EIR = Lc − Ls/Lc × 100* (2)

As well as the germination index (*GI*, Equation (3)):

*GI = (Gs × Ls/Gc × Lc) × 100* (3)

The calculations for *RGR, EIR* and *GI* were performed twice: using contaminated soil as a control (to obtain only the effect of nZVI) and using filter paper as a control (to observe interactions between the soil and nZVI). Due to the small amount of biomass produced in this experiment, no further analyses (i.e., chemical or physiological) were possible.

### 2.3. Hydroponics Experiment

Native seeds of *A. capillaris* and *F. rubra* were pregerminated in vermiculite: substrate mixture (2:3 *w*/*w*), and representative specimens were transferred after 33 days to the hydroponic standard solution (pH 6.5, 2 mM Ca(NO_3_)_2_, 2 mM KNO_3_, 1 mM NH_4_NO_3_, 1 mM KH_2_PO_4_, 0.5 mM MgSO_4_, 25 μM H_3_BO_3_, 2 μM MnSO_4_, 2 μM ZnSO_4_, 0.5 μM CuSO_4_, 0.1 μM (NH_4_)_6_Mo_7_O_24_, 50 μM NaCl and 20 μM FeEDDHA and 2 mM MES-KOH; Eh = 300 mV). A total of six plants were placed in a 1.5 L container equipped with an aerator, to provide oxygen to the roots and later to maintain the nanoparticles in suspension, which also ensured the homogeneity of the solution. During a 29-day period, the solution was renewed regularly and for the final renewal, the solution was modified as follows: the source of Fe was replaced with FeCl_2_ to avoid interference of the Fe-chelate with the absorption of nZVI [38]. From that day forward, four nZVI treatments were used: 0, 25, 50 and 100 mg·L^−1^ (T0, T25, T50, T100) with five replicates (containers) per treatment. The optimal culture conditions regarding the availability of nutrients were established in previous experiments using a geochemical/statistical approach model for nanomaterials [38]. The growing period under hydroponic conditions took place inside a germination chamber with a regime of 16:8 h light/darkness at 24/18 °C and 45% humidity.

One day after the nZVI applications, two representative roots of each specimen were marked and their growths were measured after 3, 5 and 8 days. After 40 days in the hydroponic culture, the plants were harvested, separated into roots and shoots, weighed, washed with distilled water and frozen at −18 °C for future usage. A portion of 5 g from samples obtained from control (T0) and the highest nZVI concentration (T100) were separated and lyophilized at constant weight. The lyophilized samples were kept under dry conditions until used for further analysis.

### 2.4. Analytical Procedures

Five grams of each part/plant were weighed, and the exact weight was recorded. These portions were then dried at 60 °C for 5 days, and the dry weights were measured.

The amino acid proline and the total amino acids were extracted from 1 g of tissue. The frozen samples were homogenized with a mortar and pestle in liquid N_2_ and then mixed with 15 mL of distilled water. The extraction of amino acids was performed by heating the solution (plant/water) at 100 °C for 1 h. After filtration, proline determinations were conducted using the method of Bates, et al. [39] and the total amino acid content was determined according to Lee and Takahashi [40]. The absorbances at 520 and 570 nm (proline and total amino acids, respectively) of the product were measured using a spectrophotometer with a calibration curve constructed using l-proline and glycine.

Raman spectra were measured by an InVia Raman spectrometer (Renishaw, Wotton-under-Edge, UK) equipped with a Leica confocal microscope. The instrument was calibrated to a silicon Raman band at 520.5 cm^−1^, and a 514.5 nm laser line was used with power ~1 mW at the source. We have used an objective with the magnification of 50× (Leica, Wetzlar, Germany), the exposure time was 1 s, and at each point, spectra were accumulated 15 times. The Raman spectra of lyophilized plant tissues were measured at 20 °C. The measurements were undertaken in three replicates at distinct positions within each sample. The presented spectra are averages of the three replicates. Raman data were processed using Wire 3.4 software (Renishaw, Wotton-Under-Edge, UK) and Grams AI 9.1 software (Thermo Fisher Scientific, Waltham, MA, USA).

For the elemental concentrations, including Fe, in plant samples, 0.2 g of dried material was digested in 2 mL H_2_O_2_ and 8 mL of concentrated HNO_3_ at 200 °C on a hot plate, diluted to 25 mL with deionized water, filtered through a 0.45 µm pore nitro-cellulose syringe filter and analyzed by inductively coupled plasma optical emission spectrometry (ICP OES, 720ES, Varian Inc., Palo Alto, CA, USA). For each set of samples, the reference plant material NCS ZC73018 (Bowen’s Kale; IUPAC 1979) was also analyzed.

To avoid the dilution effect in plants of different proportions, the concentration of elements was recalculated to obtain the exact amount (in mg) of each element per plant (*EP*) using Equation (4):

*EP = DW × EC* (4)

where *DW* is the plant’s dry weight (g) and *EC* is the element concentration in the plant (in mg g^−1^). The translocation factor (*TF*) was calculated as the ratio of the element concentration in the shoot to the concentration of the element in the root.

Sections of lyophilized plant roots were analyzed using scanning electron microscopy (SEM, TESCAN VEGA3XMU, Brno, Czech Republic) equipped with a Bruker QUANTAX200 energy dispersive X-ray spectrometer (EDS) (Bruker, Billerica, MA, USA), to detect the elemental distribution across the roots with and without nZVI (T100 and T0, respectively). The lyophilized sections were placed on a conductive tape and were carbon-coated before analysis.

### 2.5. Statistical Analysis

Values expressed as percentages were normalized by extracting the square root of each value and applying the arcsin function. The remaining variables were analyzed using the Shapiro-Wilk test and were normalized using a logarithmic function when needed. One-way ANOVA was performed to find the differences between treatments and Tukey’s honest significant difference method was used with a confidence level of 0.95. All statistical analyses were performed using R 3.6.1 software (The R Foundation for Statistical Computing 2018, under the GNU General Public License).

## 3. Results and Discussion

### 3.1. Germination Test

A greater number of seeds germinated in soil (Litavka and Litavka + nZVI) than under the control variant on filter paper. This could simply be due to the humidity factor: well moisturized soil can preserve more water than filter paper. Humidity is a key factor for the germination of seeds as the first step in the germination process is water absorption, which leads to the development of the first organs [41].

**Figure 1:**
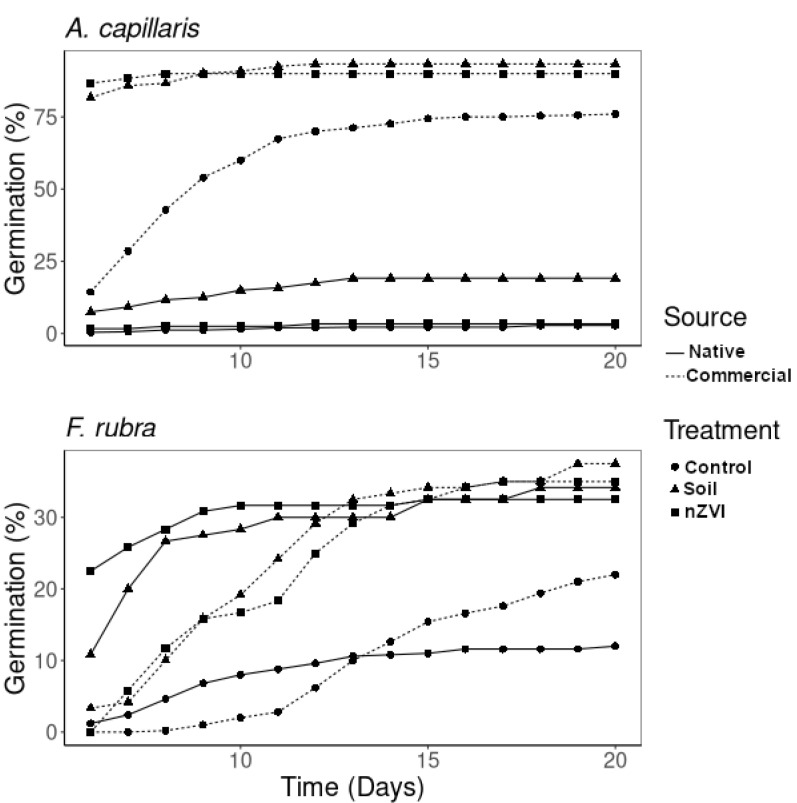
Evolution of total seed germination (%) with time (from day 6). Treatments: control, contaminated soil, and contaminated soil + 1% nZVI. Source of the seeds: native from contaminated site and commercial.

Seeds from different sources presented different responses: those obtained from the contaminated field (native) germinated at considerably lower rates than the commercial seeds (Table 2, Figure 1). Native seeds sown in untreated soil had the highest relative germination rate (*RGR* = 6.85), while, for all other treatments, the *RGR* values were below 1.3; however, values greater than 1 for all cases reflect the increased total germination in comparison to the controls. Control seeds on filter paper developed longer radicles, as expected, due to the absence of physical obstacles and from the soil itself, which naturally causes mechanical damage to the tissues. In particular, nutrition for the seedlings is provided by the endosperm [37] and, thus, nutrient acquisition is not necessary at this stage. The addition of nZVI enhanced the growth of the native seedlings of *A. capillaris* and resulted in lower elongation inhibition rates (*EIR*) in the nZVI treatment when compared with the control, which results in a negative value when compared with the soil. Negative values of *EIR* are obtained when the root is longer than for the control, while higher values represent bigger differences; the effect of nZVI by comparing treated vs. untreated soil results in *EIR* = −114, which means that the roots under treated conditions grew twice as much. This reflects the contribution of nZVI to the development of *A. capillaris* seedlings. However, in the commercial seeds, no differences were found between treated and untreated substrates (Table 2).

The application of nZVI did not significantly influence the germination of *F. rubra* seeds. Most of the native seeds of this species germinated during the first 8 days, while the commercial seeds needed 12 days (Figure 1). Although no significant differences were found between these seed types, soil treatments and seed sources (Table 2), some differences in germination times were found (Figure 1), as commercial seeds needed longer times to germinate, while native seeds treated with nZVI reached maximum germination faster. The *RGR* was higher for native than for commercial seedlings (Table 2) when compared with control, which result in *RGR* < 1 when compared with contaminated soil. Radicle lengths in the native seedlings showed no significant differences between soil treatments, but for the commercial seeds, there was an increase in the lengths under nZVI treatment when compared to the untreated soil (Table 2, EIR < 0). The effects of nZVI on *F. rubra* seedlings were more visible for the shoot lengths, as the longest shoots were found on the seedlings treated with nZVI (Table 2). In addition to germination, root elongation is one of the first visible symptoms of toxicity: root tips are sensitive to toxicity due to the influence of metal(loid)s in cell division and cell elongation [26]. This effect is visible in the *EIR* of *F. rubra*, which was higher for the commercial seedlings growing in contaminated soil (48.8), and lower for the native seedlings in the same treatment (19.4), thus, showing the adaptation of the native seeds for growth under metal(loid) contamination. Our results reflect that the application of nZVI not only presents no signs of toxicity in the early plant stages but also that for some seeds (native *A. capillaris* and commercial *F. rubra*), application of the amendment improved their development.

Germination tests are commonly used to evaluate toxicity in plants. Our results show that not only different species (*F. rubra* seedlings grew longer than *A. capillaris* ones; Table 2), but also different seed sources (probably indicating different genotypes) responded differently: native seedlings of *A. capillaris* from the contaminated site exhibited higher *EIR*. For *F. rubra*, the addition of nZVI to the soil clearly improved the growth of the commercial seedlings but had showed no significant effect on the native seedlings. Germination experiments are a quick and effective way to test for toxicity in plants; it has been proven that application of elements, such as Ni, Hg, Cd, Co, Cu and Pb, result in reductions of germination rates and seedling growth [26]. However, different plant species react differently to the same components; Munzuroglu and Geckil [42] found that applications of the same elements with the same concentrations had different effects on seed germination of wheat (*Triticum aestivum*) and cucumber (*Cucumis sativus*): wheat seeds are more sensitive to the presence of metal(loid)s, whereas cucumber seeds can cope better with their presence. Thus, testing ENPs with the potential for metal(loid) stabilization is necessary and germination tests are useful tools for evaluating this potential.

Other ENPs have also been tested in plants with phytostabilization potential and have exhibited no risk of toxicity: silver ENPs had no effect on seed germination and plant growth of *Ricinus communis* L. [27]; four kinds of Zn ENPs had no impact on the seed germination of Chinese cabbage [22]. In some cases, application of ENPs improved seedling development. A demonstration of this was found by Savithramma, et al. [43], by using silver ENPs on the seeds of *Boswellia ovalifoliolata*, which caused increased seed germination, seedling growth and hastened the germination period from 17 days in control to 8 days in treated seeds. The authors concluded that this effect could be due to the ability of the ENP to penetrate the seed coat, which could either lead to generation of new pores which allowed better nutrient flux or that ENPs may act as nutrient carriers. Research aimed at investigating the toxic effects of specific amendments and for specific plant species is an important factor for phytostabilization processes. To date, there is still very little information about the effects of nZVI on seeds and seedlings.

### 3.2. Hydroponics Experiment

**Figure 2:**
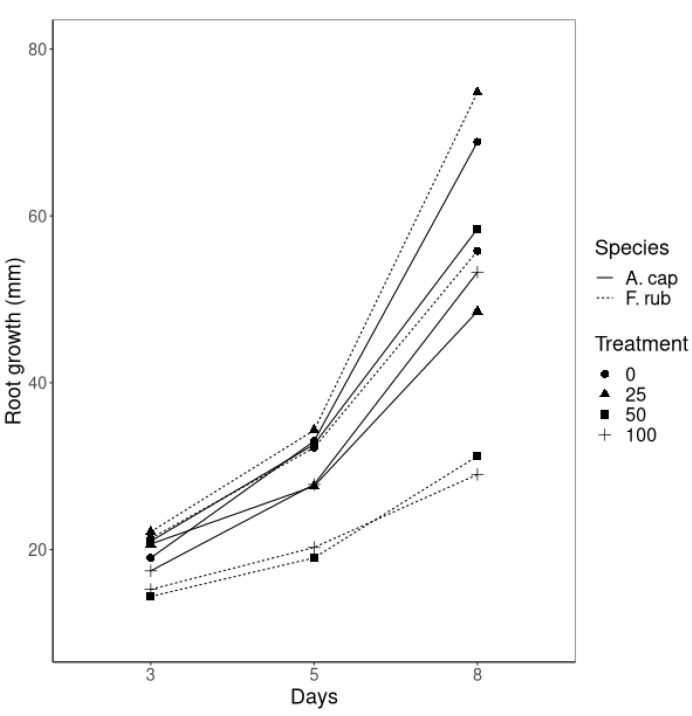
Root growth (mean value, given in mm, of 20 samples per treatment) of *A. capilaris* and *F. rubra* during the last eight days after the application of 0, 25, 50 and 100 mg nZVI L^−1^ to the hydroponic solution.

Root growth after nZVI application to the hydroponic solution exhibited small variations during the first 5 days; the main findings were for *F. rubra* development under T50 and T100, which grew less than 13 mm, while all of the roots under the other treatments grew between 11 and 59 mm (Figure 2). After 8 days with nZVI in the solution, the effects were more evident. Few studies have reported a reduction of plant growth and biomass production, due to the blockage of water transport and nutrients caused by the aggregation of ENP at the root surfaces [7,23,25]. This topic is discussed in more detail in the following sections.

The biomass production of *A. capillaris* was lower when it was not treated with nZVI (Table 3), but the roots grew longer (up to 108 mm in some replicates) than when nZVI was added to the growing medium (Figure 2). Plants treated with nZVI showed similar root biomass production and the root lengths were longer for T50, followed by T100 and T25; however, the differences were minimal (5 mm between treatment types). Regarding shoot biomass, fresh weight production followed the order: T100 > T25 > T50 > T0, while no significant differences were found for the dry weights.

*F. rubra* did not show significant differences in root biomass production (Table 3) although differences between treatments were evident in the root lengths (Figure 2). Plants treated with 25 mg L^−1^ nZVI grew the longest roots (75 mm average at final time and a maximum value of 106 mm), followed by T0 (55 mm average), while the highest concentrations of nZVI (50 and 100 mg L^−1^) developed the smallest roots.

### 3.3. Microscopic Root Observations

**Figure 3:**
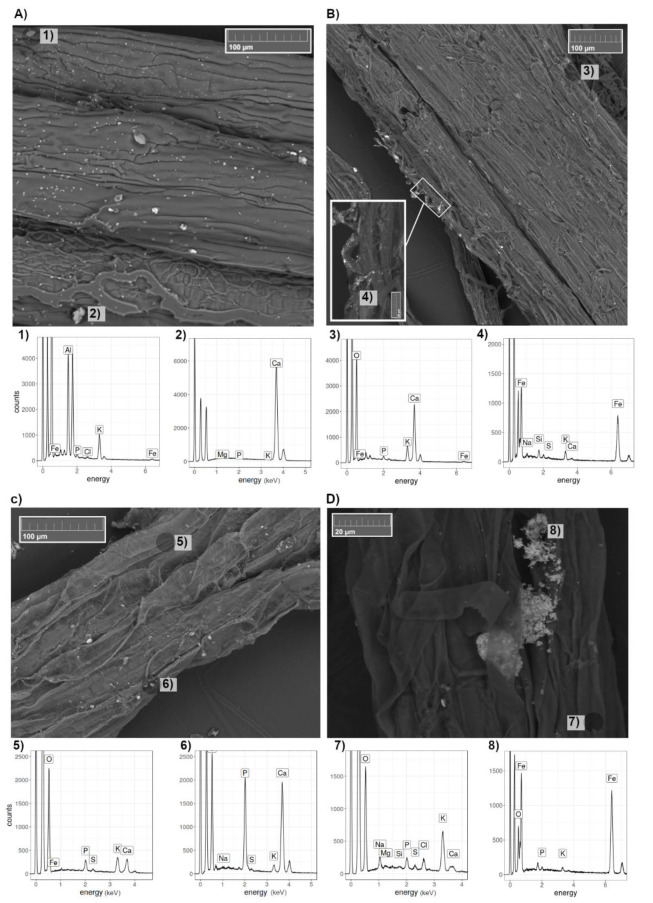
Roots of *A. capillaris* (above, (**A**,**B**)) and *F. rubra* (below, (**C**,**D**)) treated with (T100)/without (T0) nZVI (right and left, respectively) under a scanning electron microscope (SEM) with their corresponding energy dispersive X-ray spectrometer (EDS) spectra (**1**–**8**).

Roots of plants growing in the hydroponic solution treated with 0 and 100 mg L^−1^ nZVI (T0 and T100, respectively) were investigated using SEM/EDS (Figure 3) and nZVI particles were observed adhering to the roots of plants under T100 (Figure 3B,D). In agreement with this finding, nZVI particles adhering to plant roots (*Helianthus annuus* L. and *Lolium perenne* L.) have previously been identified using SEM/EDS by Vítková, et al. [44]. nZVI particles usually form aggregates, which limit their functionality for sorption and cause lower mobility of the product [45]. On the root surfaces, they create a black coating that is visible without effort [25,38], however, such coating was not found in our samples. Nevertheless, nZVI was detected under electron microscope. Observations under bright-field optical microscopy in previous studies [28] showed that the nZVI particles aggregated outside of the cell walls. It has been hypothesized that such aggregation could be the reason for the reduction in water movement to the plant and thus, nutrient uptake, however, this has not been clearly proved yet [25,38].

Our observations of *F. rubra* roots under T0 also showed several particles of calcium phosphate (Figure 3(C6)) adhering to the roots. These particles could possibly be aggregated due to the presence of Ca(NO_3_)_2_ in the hydroponic solution, which is known to control *p* release in solutions, due to its oxidative effect on reduced substrates [46].

### 3.4. Physiological Parameters

**Figure 4:**
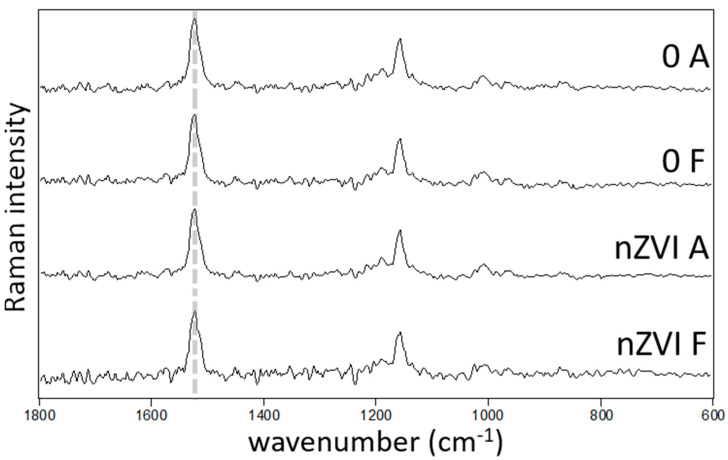
Raman spectra of *A. capillaris* (A) and *F. rubra* (F) untreated (0) and treated with 100 mg L^−1^ nZVI, presented as an average from three measurements at distinct zones of the leaf. The spectral features correspond to carotenoids; the v1(C=C) band position (dashed line), which may reflect changes in the carotenoid structure remains the same (1523 cm^−1^) among the measurements.

For the stress markers analyzed in the plants at the end of the hydroponic experiment, no significant differences were found between treatments (Table 3, Figure 4). Proline is an amino acid that plays several roles in plants, such as biosynthesis of proteins, the scavenging of reactive oxygen species, the protection of photosystems and regulating cellular pH [47]. Proline accumulation often occurs in the presence of elevated metal(loid)s concentrations in plants [48], e.g., Ullah, et al. [49] found a strong correlation between Pb, Cr and proline concentrations in plant tissues. Proline concentrations can also be indicators of nutrient deficiency and of salt and/or drought stress [30,31]. Our results showed no differences in the levels of proline and total amino acids (Table 3) in plant tissues with increasing nZVI concentrations, which can indicate no toxicity. The carotenoid composition and structure also show no differences between treatments (Figure 4). Carotenoids, non-enzymatic antioxidant pigments, are involved in the protection of chlorophyll production during oxidative stress; other studies have found differences in the carotenoid activity in plant species of the *Poaceae* family, in the presence of soil contamination, compared with no contamination or amended soils [32,33,50]. Our results correlate well with other research, which has found no or little effects of nZVI on the regular physiological functions of different plant species: Concentrations of 0, 250 and 1000 mg kg^−1^ had no impact in seed germination, biomass production or root and shoot lengths of *Oryza sativa* [24]; likewise, 5 and 50 g L^−1^ had no significant effects on the germination index, seedling elongation or dry weight of *Lepidium sativum*, *Sorghum saccharatum* and *Sinapis alba* [28].

**Figure 5:**
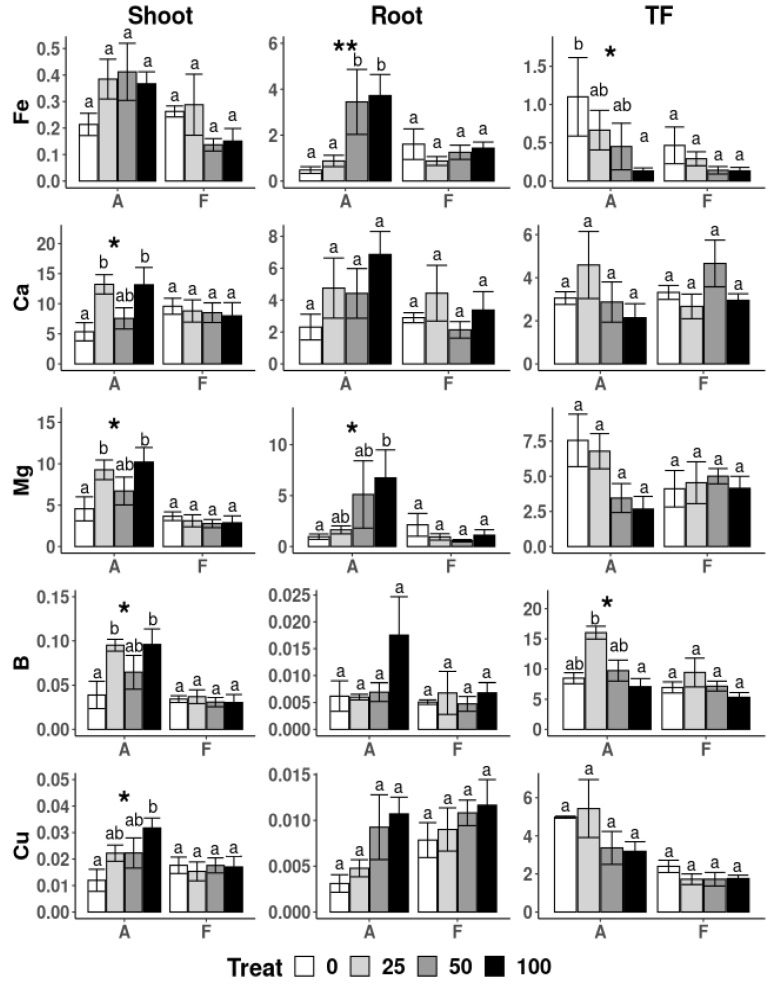
Translocation Factor (TF) and uptake of elements (g) in shoot and root biomass of *A. capillaris* (A) and *F. rubra* (F) at the end of the hydroponic experiment for the different treatments (e.g., 0, 25, 50 and 100 mg nZVI L^−1^). For the one-way ANOVA * *p* < 0.05, ** *p* < 0.01, no asterisks for *p* > 0.05. Different letters above the bars represent significant differences according to Tukey’s test at *p* < 0.05.

*A. capillaris* plants showed differences in the Fe concentrations in the roots, which were very low in the plants exposed to the lowest concentration of nZVI (25 mg·L^−1^), and were similar to those observed when no nZVI was applied (Figure 5). Significantly higher Fe concentrations were observed at higher nZVI doses. Consequently, a decrease in the translocation factor (TF) with an increase in nZVI in the growing solution was observed. Iron is required for various cellular processes in plants, including respiration, chlorophyll biosynthesis and photosynthesis and serves as a cofactor for enzymes involved in electron or oxygen transfer [51]. However, the mechanisms of uptake and accumulation of nZVI into the plant tissues is still uncertain; its movement within the plant is unknown. Copper usually binds well with Fe and, thus, its distribution in plants is similar, with lower levels in plants with the lowest nZVI concentrations, and increasing levels with higher doses of the amendment, which results in a decrease of TF, with increases in nZVI concentrations in the hydroponic/growing solution.

**Figure 6:**
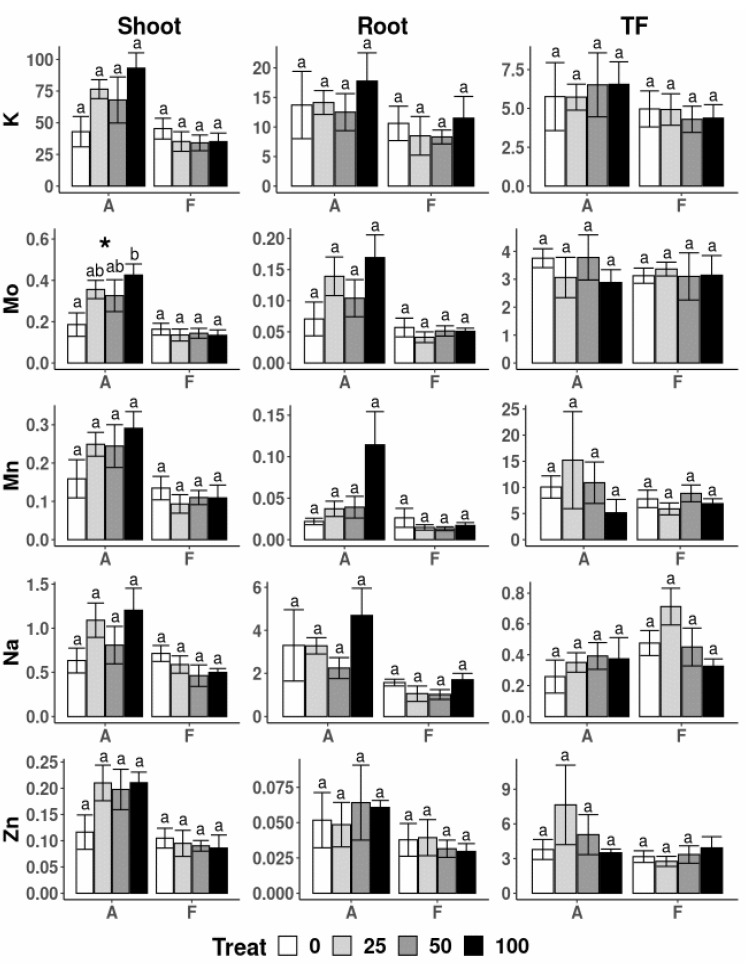
Translocation Factor (TF) and uptake of elements (g) in shoot and root biomass of *A. capillaris* (A) and *F. rubra* (F) at the end of the hydroponic experiment for the different treatments (e.g., 0, 25, 50 and 100 mg nZVI L^−1^). For the one-way ANOVA * *p* < 0.05, ** *p* < 0.01, no asterisks for *p* > 0.05. Different letters above the bars represent significant differences, according to Tukey’s test at *p* < 0.05.

The application of increasing concentrations of nZVI also affected the uptake by *A. capillaris* of some major nutrients (Figure 5 and Figure 6). The Ca, Mg and B contents in the shoots of plants treated with 25 and 100 mg L^−1^ were significantly different than those in plants with no nZVI application. A different effect was observed in the roots of this species, which showed great variability and only the Mg content at the highest nZVI dose showed significant differences compared to those in the control (no nZVI) solution. The TF of B was highest in plants treated with 25 mg nZVI L^−1^ and means that, with this particular treatment, an increase in B transport to the upper plant parts took place. Other nutrients, including K, Mo, Mn, Na and Zn, were found in the lowest amount in the shoots of *A. capillaris* treated without nZVI and no other differences were found between treatments (Figure 6).

Regarding *F. rubra* plants, no effects of the nZVI applications were found in the physical, physiological or chemical parameters measured (Table 3, Figure 5). Consistent with these findings, Martínez-Fernández and Komárek [23] reported no significant effects on the growth of *Solanum lycopersicum* under hydroponic conditions with nZVI addition. Nevertheless, in the present experiment, there was evidence of aggregation of nZVI particles onto the root surfaces of *F. rubra* (Figure 3D). This suggests that *A. capillaris* possesses mechanisms that are able to assimilate nZVI more so than *F. rubra*. One possibility could be the formation of iron plaque at the root levels. Research shows that the formation of iron plaque on the roots induces variations in the movement of nutrients and trace elements within the plant, e.g., increases of P, S and Mg uptake [52,53], which could explain the increase of Mg and Fe contents in the roots of *A. capillaris* at higher nZVI concentrations (Figure 5). Most of the information available on iron plaque formation comes from experiments performed on rice and other well-known halophytes or flood-resistant species. Our seeds were collected from a grassland dominated by *A. capillaris* that is regularly flooded, which suggests that this species and, in particular, this genotype could potentially be adapted to such conditions. Furthermore, other species of the family *Poaceae* have also been shown to develop iron plaque, such as *Paspalum urvillei* and *Setaria parviflora*, which grow in sand with excesses of iron and developed layers of Fe oxyhydroxide (ferrihydrite) in the cell walls and vacuoles, which were detected only by synchrotron µXANES analysis [54]. The evidence suggests that one of the mechanisms of *A. capillaris* to cope with nZVI could be the formation of iron plaque, however, further investigation is necessary to evaluate this concept.

The two plant species have been found to grow in close association in multicontaminated soil, and our previous studies have shown evidence of their potential as phytostabilizing plants [34]. The differences in their responses to the application of nZVI highlights the importance of biodiversity in phytoremediation projects, and supports the possibility of nZVI applications as amendments for assisted phytostabilization using *A. capillaris* and *F. rubra* associations.

## 4. Conclusions

Two species of the family *Poaceae*, which grow in close association in a contaminated site, responded differently to the application of nZVI. In general, the two species exhibited no symptoms of toxicity under nZVI application, but rather received support for the development of certain characteristics. Particularly for *A. capillaris,* this is reflected by an increase of biomass production and root growth. The diversity in the behavior and response of each plant species suggests that the use of *A. capillaris* and *F. rubra* in association could be a good strategy for the successful revegetation of contaminated soils, by ensuring the survival of numerous individuals of the species when assisted by nZVI, and depending on the interactions and responses of the contaminant(s). However, this is beyond the scope of this work and it requires further investigation describing robustly the plant communities—nZVI—contaminant(s) relationships.

Additionally, our findings suggest that nZVI could be assimilated by the plants, although in different proportions, depending on the species. Based on our results, we hypothesize that *A. capillaris* has mechanisms that can better assimilate nZVI than *F. rubra*. However, such mechanisms are still unknown and require additional investigation. Further research to study nZVI availability and mobility within plants and iron plaque formation in the roots, as well as the direct interactions between plants-nZVI-contaminants (e.g., in hydroponic systems) can provide valuable insights to the development of strategies that use *Poaceae* species for the phytostabilization of contaminated soils assisted by nZVI.

## Figures and Tables

**Table 1:** Characteristics of the contaminated soil used in the germination experiment. Data are presented as the mean values and standard deviations of the bulk soil and rhizospheric soil of *A. capillaris* and *F. rubra* growing in association. Concentrations of elements are all given in mg·kg^−1^.

Details	^1^ Bulk Soil	^2^ Rhizosphere
Clay	5%	-
Silt	20%	-
Sand	75%	-
pH-H2O	5.95 ± 0.01	5.62 ± 0.27
pH-KCl	5.14 ± 0.03	5.37 ± 0.37
K	6583 ± 293	5534 ± 716
Ca	1778 ± 104	2463 ± 655
S	490 ± 6	410 ± 154
Cu	71.9 ± 3.0	84 ± 12
Mn	4276 ± 28	1857 ± 36,728
Fe	37,408 ± 159	23,930 ± 2684
Zn	4002 ± 55	2910 ± 493
Pb	3539 ± 30	2796 ± 425
As	296 ± 5	237 ± 48
Cd	39 ± 0.1	36 ± 6

^1^ Data obtained from Vítková, et al. [36]. ^2^ Data obtained from Teodoro, et al. [34]. More details about the site can be found in Ettler, et al. [35].

**Table 2:** Mean values ± standard deviation of the seedling characteristics after the germination experiment. Total germination (%), root and shoot length (cm), relative germination rate (RGR), elongation inhibition rate (EIR) and germination Index (GI). Treatments: control (C), contaminated soil, and contaminated soil + 1% nZVI (nZVI). The calculations of RGR, EIR and GI are provided for both control contaminated soil and control without soil and denoted as “Soil + nZVI” and “nZVI effect”, respectively. Source of seeds: native from contaminated site and commercial. Different letters represent significant differences under Tukey’s honest significance test for *p* < 0.05.

Source	Treat	Germ	Root	Shoot	RGR	EIR	GI
		(%)	(cm)	(cm)			
		*Agrostis capillaris*					
Native	C	^a^ 2.8 ± 2.1	^c^ 2.5 ± 1.7	^b^ 1.6 ± 0.5	1.00	0.00	100
	Soil	^b^ 19.2 ± 3.8	^a^ 0.3 ± 0.2	^a^ 1 ± 0.8	6.85	90.7	63.4
	Soil + nZVI	^a^ 3.3 ± 4.1	^b^ 0.5 ± 0.38	^b^ 1.8 ± 0.7	1.19	80.1	23.6
	nZVI effect				0.17	−114	37.3
Commercial	C	^c^ 76 ± 6.7	^c^ 3.7 ± 1.1	^b^ 2 ± 0.5	1.00	0.00	100
	Soil	^d^ 91.7 ± 7.5	^b^ 0.8 ± 0.3	^c^ 3.5 ± 0.9	1.21	78.3	26.1
	Soil + nZVI	^d^ 90 ± 10	^b^ 0.8 ± 0.3	^c^ 3.2 ± 0.7	1.18	79.0	24.9
	nZVI effect				0.98	2.89	95.3
		*Festuca rubra*					
Native	C	^a^ 12.0 ± 2.8	^bc^ 3.0 ± 1.7	^a^ 4.2 ± 4.2	1.00	0.00	100
	Soil	^b^ 34.2 ± 15.3	^abc^ 2.4 ± 1.4	^c^ 6.9 ± 2.3	2.85	19.4	229.6
	Soil + nZVI	^b^ 32.5 ± 12.9	^ab^ 2.0 ± 0.9	^c^ 7.6 ± 2.3	2.71	32.2	183.6
	nZVI effect				0.95	15.9	79.9
Commercial	C	^a^ 22.0 ± 8.5	^c^ 3.3 ± 1.3	^a^ 3.5 ± 1.2	1.00	0.00	100
	Soil	^b^ 37.5 ± 19.2	^a^ 1.7 ± 1.0	^ab^ 4.6 ± 2.1	1.70	48.8	87.3
	Soil + nZVI	^b^ 35 ± 11.4	^abc^ 2.4 ± 0.4	^bc^ 6.9 ± 1.3	1.59	27.8	114.8
	nZVI effect				0.93	−40.9	131.4

**Table 3:** Plant characteristics at the end of the hydroponic experiment. Treatment (T) expresses mg of nZVI per L of hydroponic solution. ANOVA was performed for shoot and root separately. Different letters represent significant differences when using Tukey’s honest significance test test *p* < 0.05.

T	Part	Fresh Weight	Dry Weight	Aminoacids	Proline
		(g)	(g)	μM g^−1^	μM g^−1^
*A. capillaris*					
0	Shoot	^a^ 8.72 ± 1.7	^a^ 2.17 ± 1.56	^a^ 147 ± 44	^a^ 5.21 ± 2.48
25		^bc^ 21.6 ± 4.4	^a^ 4.13 ± 0.85	^a^ 102 ± 64	^a^ 4.02 ± 2.36
50		^ab^ 12.9 ± 5.0	^a^ 3.48 ± 2.12	^a^ 167 ± 58	^a^ 5.42 ± 1.57
100		^c^ 26.3 ± 9.4	^a^ 5.08 ± 1.58	^a^ 155 ± 36	^a^ 3.4 ± 0.88
0	Root	^a^ 5.11 ± 1.1	^a^ 0.43 ± 0.09	^a^ 72 ± 16	^a^ 2.71 ± 2.68
25		^b^ 16.8 ± 3.4	^ab^ 1.42 ± 0.2	^a^ 76 ± 10	^a^ 4.53 ± 2.11
50		^ab^ 15.9 ± 10.2	^ab^ 1.33 ± 1	^a^ 74 ± 13	^a^ 4.44 ± 2.94
100		^b^ 19.4 ± 6.4	^b^ 1.67 ± 0.48	^a^ 78 ± 23	^a^ 3.86 ± 2.53
*F. rubra*					
0	Shoot	^a^ 12.21 ± 5.1	^a^ 2.16 ± 0.76	^a^ 161 ± 36	^a^ 5.93 ± 2.77
25		^a^ 9.63 ± 4.7	^a^ 1.87 ± 0.8	^a^ 159 ± 34	^a^ 4.31 ± 1.47
50		^a^ 8.88 ± 3.3	^a^ 1.78 ± 0.61	^a^ 158 ± 58	^a^ 3.21 ± 1.08
100		^a^ 10.05 ± 5.1	^a^ 2 ± 1.04	^a^ 142 ± 30	^a^ 4.42 ± 0.85
0	Root	^a^ 9.81 ± 3.7	^a^ 0.75 ± 0.26	^a^ 49 ± 6	^a^ 3.48 ± 2.73
25		^a^ 8.43 ± 4.6	^a^ 0.7 ± 0.32	^a^ 76 ± 47	^a^ 3.46 ± 2.62
50		^a^ 8.40 ± 2.9	^a^ 0.72 ± 0.27	^a^ 46 ± 13	^a^ 2.77 ± 2.86
100		^a^ 8.71 ± 5.6	^a^ 0.77 ± 0.48	^a^ 66 ± 34	^a^ 3.63 ± 2.8
